# Sensitivity of proxies on non-linear interactions in the climate system

**DOI:** 10.1038/srep18560

**Published:** 2015-12-21

**Authors:** Johannes A. Schultz, Christoph Beck, Gunter Menz, Burkhard Neuwirth, Christian Ohlwein, Andreas Philipp

**Affiliations:** 1 Department of Geography, University of Bonn, 53115 Bonn, Germany; 2 Institute of Geography, University of Augsburg, 86159 Augsburg, Germany; 3 DeLaWi Dendro Lab Windeck, 51570 Windeck, Germany; 4 Hans-Ertel-Centre for Weather Research, Meteorological Institute, University of Bonn, 53121 Bonn, Germany

## Abstract

Recent climate change is affecting the earth system to an unprecedented extent and intensity and has the potential to cause severe ecological and socioeconomic consequences. To understand natural and anthropogenic induced processes, feedbacks, trends, and dynamics in the climate system, it is also essential to consider longer timescales. In this context, annually resolved tree-ring data are often used to reconstruct past temperature or precipitation variability as well as atmospheric or oceanic indices such as the North Atlantic Oscillation (NAO) or the Atlantic Multidecadal Oscillation (AMO). The aim of this study is to assess weather-type sensitivity across the Northern Atlantic region based on two tree-ring width networks. Our results indicate that nonstationarities in superordinate space and time scales of the climate system (here synoptic- to global scale, NAO, AMO) can affect the climate sensitivity of tree-rings in subordinate levels of the system (here meso- to synoptic scale, weather-types). This scale bias effect has the capability to impact even large multiproxy networks and the ability of these networks to provide information about past climate conditions. To avoid scale biases in climate reconstructions, interdependencies between the different scales in the climate system must be considered, especially internal ocean/atmosphere dynamics.

Dense proxy networks are often used to reconstruct temperature^1^,^2^,^3^,^4^, droughts^5^,^6^ or precipitation^7^,^8^. Due to the interrelation of processes at different spatiotemporal scales in the climate system (micro, local, meso, synoptic and global scale), it is even possible to use proxies – such as tree rings – which react to micro/local climate conditions, to reconstruct phenomena on the global scale of the climate system such as the Pacific Decadal Oscillation (PDO)^9^, or the North Atlantic Oscillation (NAO)^10^. These indices condense complex spatial and temporal climate/ocean variability in a simple measure which is sometimes not easy to relate with local climate conditions^11^. To describe the state of the atmospheric circulation more comprehensively than via circulation indices, circulation- or weather- type classifications can be applied. A number of authors have shown that the use of weather-type data can improve the understanding of climate/growth relationships and past climate conditions^12^,^13^,^14^. It is a common approach to describe and analyse weather and climate conditions by applying weather-type classifications which group the atmospheric circulation states based on multivariate information given on the metrical scale in an input dataset, such as daily pressure fields, into distinct types, so-called circulation- or weather-types^15^. Weather-types are the leading factor for local and regional climate conditions^16^.

Much effort has been made to improve climate reconstructions by including larger multiproxy datasets for both hemispheres and by the comparison of results^3^,^17^,^18^. To reduce uncertainties and to enhance the quality of climate reconstructions the used methods^19^ or e.g. the tree-ring standardisation procedures^20^,^21^ – to remove age-related growth trends^2^– are constantly improved.

Besides these efforts for several locations temporally unstable climate/growth relationships, a reduced sensitivity of tree-rings to single climate elements^22^,^23^ were reported. In this context, the divergence problem^24^ or the reported spectral biases^25^ in proxy networks are widely discussed.

In addition to these issues our results demonstrate that phenomena operating on the synoptic to global scale (here NAO, AMO) of the climate system can affect the climate sensitivity of tree-rings for phenomena in subordinate levels of the climate system (here weather-types, meso- to synoptic scale). This scale bias effect can be generally defined as a reduced climate sensitivity of proxies for phenomena in subordinate levels of the climate system, caused by nonstationarities in superordinate scales of the climate system. For instance, a reduced climate sensitivity of proxies for phenomena in the micro scale of the climate system can also be referred to as scale bias if it is caused by nonstationarities in the meso-, synoptic-, or global scale of the climate system.

The scale bias effect has the capability to severely impact climate reconstructions^18^. Reconstructions of superordinate scales phenomena such as NAO^1^,^10^, AMO^1^ or PDO^1^,^26^ are expected to be more robust, due to the low-pass filtering effect when regarding larger scale phenomena.

In this study, we utilise nine weather-/circulation-type classifications in combination with two tree-ring datasets to demonstrate the scale bias effect.

## Methods

### Classification approaches for the determination of circulation types

The study is based on nine weather-type classifications, the subjective (manual) Hess Brezowsky (H/B)^27^ classification which defines 29 weather types (so called “Großwetterlagen”) and eight objective, computer-assisted, circulation-type classifications, encompassing the spatial domain 54 °W to 70 °E/30 °N to 76 °N.

The weather-type classifications for the period 1871–2010 have been derived by applying the cost733class classification software^28^ to daily (12 UTC) 2° by 2° gridded 1000 hPa and 500 hPa geopotential height data. These data were accessed directly through the NOAA-CIRES 20^th^ Century Reanalysis data archive^29^. As different weather-type classifications may reflect varying aspects of atmospheric circulation dynamics, two classification approaches were selected – GWT (Großwettertypen)^30^,^31^; DKM (*k*-means clustering) –^15^. Both classification approaches were conducted in two variants, producing 18 and 27 classes (types) respectively. Consequently, a total of eight classifications were computed – two pressure levels, two classification methods, two variants (18 or 27 classes).

The first classification approach is the Grosswettertypes (GWT) or Prototype classification^30^,^31^. GWT utilises the cyclonicity and main direction of large-scale air flows to arrange cases (daily geopotential height fields) into classes/types. These classes are predefined according to the Hess Brezowsky (H/B) classification^27^. The second classification approach (DKM)^15^ is based on non-hierarchical k-means clustering e.g.^32^. DKM utilises most dissimilar cases (daily geopotential height fields) to determine the starting partition^15^,^33^ for the subsequent iterative reassignment of cases to classes. This procedure continues until no further improvement (in terms of reduction of the within-cluster variances) can be achieved. These two classification approaches were chosen as previous studies e.g.^34^ have shown their particular ability to resolve surface climate variations in Europe.

### Tree-ring data and detrending

Two tree-ring datasets were used to determine the influence of scale bias effects in tree-ring networks. Tree-ring dataset-1 is based on 21 beech and 29 oak chronologies which were taken from an already published tree-ring width network^12^,^35^,^36^. To preserve high frequency signals and to maintain interannual variability, the synchronised raw measurements were detrended by calculating residuals from 32-year cubic smoothing splines with a 50% frequency-response cutoff^37^. This detrending procedure removes tree age-related growth trends as well as variability which is related to interdecadal and multidecadal time scales.

To calculate site chronologies a bi-weight robust mean was used. The values of the statistical parameters, expressed population signal (EPS)^38^ which indicates how well the site chronology estimates a theoretically infinite population and interseries correlation (Rbar)^39^– averaged correlation between tree-ring series – are shown with additional meta information in Supplementary Table S1. EPS and Rbar values were calculated for 30-year segments lagged by 15 years. Only averaged values are shown but in all investigated segments the EPS values constantly range above the commonly applied threshold of 0.85^38^. All chronologies cover the complete investigation period 1891–1990 (Supplementary Table S1). The tree ring dataset includes only Central European tree-ring width chronologies (48–52 °N/7 °E-11 °E) and showed common climate relationships.

We used dataset-2 to verify the results derived from dataset-1. Dataset-2 is a subsample of the dataset gathered by Babst *et. al.*^36^, has a larger spatial domain (30–70 °N/10 °W-40 °E), includes 726 chronologies and covers most of Europe and North Africa. As previously stated it is necessary to remove the biological age trend^2^. They used 32-year cubic smoothing splines with a 50% frequency-response cutoff and additionally, in contrast to dataset-1, an adaptive power transformation^40^ was applied before calculating residuals from the splines. This was done to stabilise the heteroscedastic variance structure in this large dataset^36^. Besides changes regarding tree-ring detrending, the procedure settings were slightly changed by shifting the investigation window by 10 years (1881–1980) which leads to completely different calibration years used for the weather-type procedure^12^. This shift also allows the use of a greater number of chronologies (a total of 726). The common period between the two tree-ring dataset is therefore 1891–1980, which enables to investigate the complete period of unstable climate growth relationships described in the result chapter with both datasets.

### Atmospheric circulation tree-ring index (ACTI) and climate datasets

The atmospheric circulation tree-ring index (ACTI)^12^ is used to assess the weather-type sensitivity in the tree-ring width networks. ACTI allows to link nominal scaled weather-type data with metric proxy data. The procedure to calculate ACTI enables investigation of weather-type signals in tree-ring chronologies and is applicable to different weather-type classifications and is explained in the following. Since a single weather-type can cause different weather condition patterns in respect to the season, it is useful to perform a separate simulation for each season. Our analysis is focused on the spring season due to the interrelation found within climate datasets (discussed in the Result section).

An ACTI time series is computed for each weather-type classification and for every site chronology.

The values of the ACTI time series are defined as springtime sums of the weighted weather-type frequencies during the period 1891 to 1990 (for tree-ring dataset-1) and 1881 to 1980 (for tree-ring dataset-2), respectively. As an example, 27 weather-type weights are needed to calculate ACTI for a weather-type classification with 27 classes/types. These weights are computed, based on a Monte Carlo simulation with 1 million simulation runs. In each Monte Carlo run a random number set which contains 27 (or 18, or 29) normal distributed random numbers is used to compute a randomly weighted weather-type index. In each run 60 discontinuous calibration years are selected and different measures of coherence are computed between all tree-ring chronologies and the weather-type index. Since several hypotheses are tested (1 million simulation runs), the experimentwise error rate is larger than the individual error rate^41^. For the necessary multiple comparison adjustment the Sequential Goodness of Fit (SGoF)^42^ metatest was used, because its statistical power increases with the number of tests performed. Besides SGoF the procedure has several (in total 5) selecting steps e.g. to reduce the influence of outliers, to exclude signals which are only related to a single site chronology and to exclude “poor performing” random number sets. Finally, according to the law of large numbers for each site chronology, a minimum number of random number sets is required to calculate the weather-type weights. Due to the design of the procedure, weather types which have a small influence on tree-ring growth will receive a weight value that approaches or reaches zero. All tree-ring chronologies –including those with a weak or not significant weather-type response contribute (at least indirectly) to the determination of weather-type signals in the tree-ring width network utilised here. Based on the weather-type weights for each tree-ring chronology, ACTI time series are computed, which are always positively correlated with the corresponding tree-ring chronology. For tree-ring dataset-1 the observed strong coherence between the ACTI time series and the tree-ring chronologies (see Supplementary Fig. S3) enables to compute a single ACTI time series and a mean tree-ring width chronology for each of the nine weather-type classifications.

For tree-ring dataset-2 this proceeding is not useful due to the vast spatial domain and the large number of tree species (Supplementary dataset S2 Inventory). In consequence a principal component analysis (PCA) e.g.^43^,^44^ was conducted for grouping the ACTI time series. For each weather-type classification based on the ACTI time series (computed for every chronology) the first principal component is computed. For both tree-ring datasets we did not use the grouping algorithm implemented in the procedure which allows to investigate spatiotemporal growth patterns because this study is focused on the investigation of common large-scale climate signals.

Additionally, we used the following climate datasets to investigate climate signals in the ACTI time series and tree-ring chronologies: The high-resolution (0.25° by 0.25°) gridded E-Obs 10 dataset^45^ was used for precipitation and maximum temperature. The GISS Surface Analysis dataset^46^ (250 km resolution) was used for temperature. Pressure data for the period of 1948 to present were acquired from the NCEP/NCAR Reanalysis 1 project^47^. Further two NAO datasets^48^ – NAO_Azores and NAO_Gibraltar – were used. The Hadley Centre Sea Surface Temperature dataset^49^ was applied to calculate SST_N and SST_S.

## Results

### ACTI time series and their climate information

**Figure 1:**
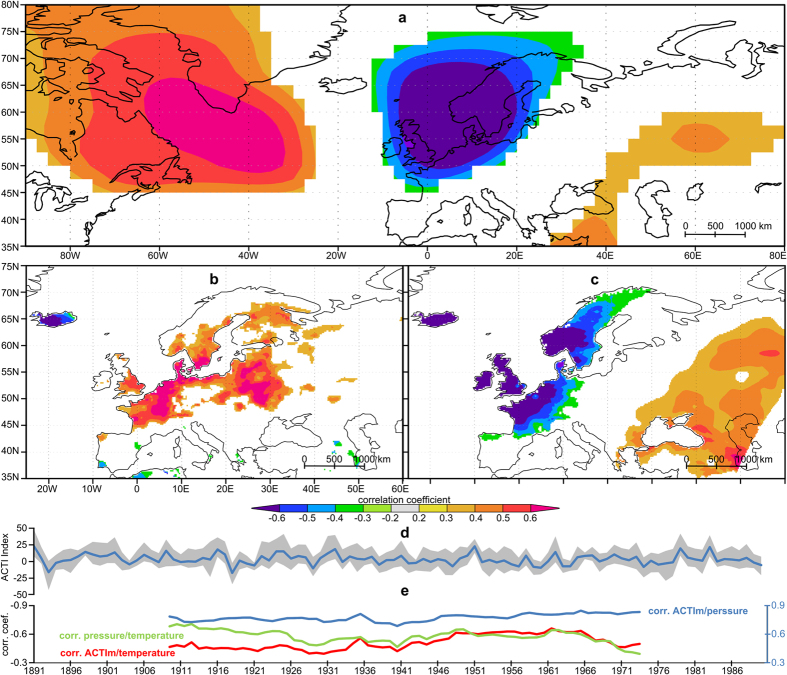
Spatial and moving correlation computed between gridded climate datasets (a-c, e), and ACTIm (d). (**a**) NCEP/NCAR Reanalysis 1, pressure^47^ (500 hPa), (**b**) E-OBS 10, precipitation^45^. (**c**) E-OBS 10, maximum temperature^45^. (**d**) ACTIm (blue) with the fluctuation range (gray) of all ACTI time series. (**e**) moving correlation analyses in 35-year window between ACTI and climate datasets (20th Century Reanalysis data pressure^29^ and GISS Surface Analysis temperature^46^). The maps were generated with the Climate Explorer^59^ web application a service of the Royal Netherlands Meteorological Institute and arranged with Inkscape. Coloured areas reflect significance (*p* < = 0.05). Pearson correlation coefficients were computed based on the 1951–1990 period common among the datasets (**a**–**c**). All calculations (**a**–**c**, **e**) are based on high pass filter time series (year-on-year differences).

Based on tree-ring dataset-1 for each weather-type classification an ACTI time series for the period 1891 to 1990 was computed. Additionally, a mean curve (ACTIm) was derived from the nine resulting ACTI time series. Therefore the results are based on a weather-type ensemble to ensure that the detected instability between weather-types and tree-ring chronologies is not a statistical artefact in the weather-type data. Also the temporal stability of the relationships between ACTI and climate data and the spatial structure of the correlation patterns are investigated. In Fig. 1 the highly significant correlation coefficients (Pearson correlation coefficient; *p* < 0.001) computed between ACTIm and gridded climate datasets indicate that the tree-ring network (dataset-1) used in this study is sufficiently large enough in order to capture large-scale climate signals across the Northern Atlantic region. As stated previously, ACTI is always positively correlated with the corresponding tree-ring chronologies^12^. The negative correlation with atmospheric pressure (*r* < -0.6, Fig. 1a) is consistent with the positive correlation pattern found for precipitation (*r* > 0.6 Fig. 1b) as well as the negative maximum temperature correlation pattern (*r* < -0.6 Fig. 1c). Temperature and precipitation are the dominant growth controlling factors for the used tree-ring width network. Similar correlation pattern were found between the mean tree-ring width chronology and gridded climate datasets (see Supplementary Fig. S2). The patterns presented in Fig. 1 reflect the typical track of low-pressure systems and their associated precipitation fields. The pattern depicted in Fig. 1a are consistent and similar with correlation patterns for pressure for all ACTI time series presented in Supplementary Fig. S1. The results indicate common climate signals within the ACTI time series (see also Supplementary Fig. S3b).

To investigate the temporal stability of the statistical coherence between climate datasets and ACTI, the region showing the strongest statistical coherence between ACTIm, pressure and temperature (Fig. 1a,c) is used (55–65 °N/5-15 °E). For both climate elements long datasets covering the full length of the ACTI time series with a good spatial resolution are available. For the described region we computed a spatial mean curve based on the GISS Surface Temperature Analysis dataset^46^ (250 km resolution) and the 20th Century Reanalysis dataset^29^ (500 hPa pressure, resolution 2°-2°). The correlation coefficients, computed for a 35-year moving window between them, indicate no systematic instabilities (Fig. 1e). The highest fluctuation range occurs between pressure and temperature followed by ACTIm and temperature. The lowest fluctuation range and the strongest statistical coherence is between ACTIm and pressure. The results shown in Fig. 1, in combination with Supplementary Fig. S1, indicate that the ACTI time series have a stable coherence with climate datasets and are able to capture atmospheric variability.

### The scale bias effect

**Figure 2:**
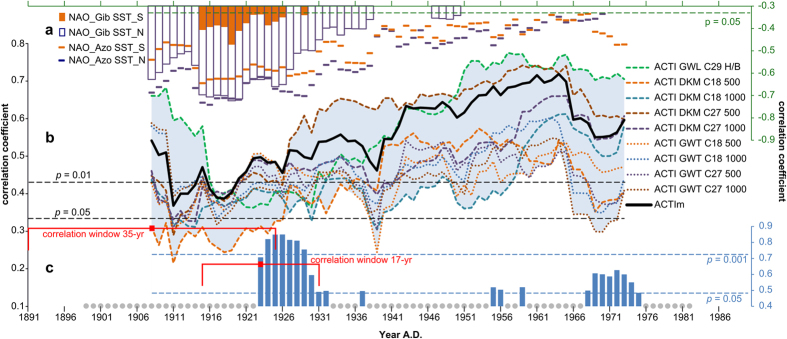
Moving correlation analysis (**a**) between two different Sea Surface Temperature^49^ (SST_N, SST_S) and NAO^48^ (NAO_Gib = NAO Gibraltar, NAO_Azo = NAO Azores) datasets, (**b**) between ACTI time series and tree-ring chronologies and (**c**) between the mean SST time series and the mean tree-ring chronology. For (**a**) and (**b**) a 35-yr correlation window and for (**c**) a 17-yr window is used to get a sufficiently fine resolution. In (**a**) and (**c**) only significant correlation coefficients *p* < = 0.05 are displayed. Grey circles in (**c**) on the X-axis indicate correlation windows with insignificant correlation coefficients. For (**a**–**c**) the Pearson correlation coefficient is used. SST_N defined as average 15°–50 °W/40°–60 °N, and SST_S as average 15°–70 °W/0°-30 °N.

In contrast to the results above, the moving correlation analysis (35-year window) between ACTI time series and tree-ring chronologies indicates synchronous fluctuations in weather-type sensitivity (Fig. 2b). Additionally, the variability in and among the curves plotted in Fig. 2b is high, exemplified by the large range between the correlation coefficients (*r* = 0.36 to *r* = 0.73) for the year 1951 (correlation window 1934–1968). The black curve in Fig. 2b displays the moving correlation between ACTIm and the mean tree-ring chronology. This curve demonstrates the advantages of the weather-type classification ensemble in yielding reduced variability and stable statistical relationships (*p* < 0.05).

In the beginning of the time series (1908) nine out of ten curves indicate a significant relationship between ACTI and tree-ring chronologies. For eight curves *p* <= 0.01 and for one curve 0.01< *p* < 0.05. All curves especially the objective weather-type classifications show a decline in statistical relationship in 1911. The GWL C29 H/B shows another sharp decrease in 1916. The lowest variability between the curves shown in Fig. 2b is in the period 1916–1927. The observed changes in weather-type sensitivity of the tree-ring network as well as the reduced variability between the curves, is an indication for a common trigger in the period 1911–1927. In contrast to 1908 all curves especially in 1911 and 1918, show a reduced weather-type sensitivity (*p* > 0.01). After 1918 a positive trend is observable until 1965. Towards the end of the time series the statistical relationship between ACTI time series and tree-ring chronologies decreases again, especially for the objective weather-type classifications.

A spatial correlation analysis between gridded Hadley Centre SST data and the NAO index shows negative correlation coefficients (*p* < 0.01) in SST_N and SST_S areas (not shown, areas defined in the figure caption of Fig. 2) in the beginning of the 20^th^ century. The moving correlation analysis between the averaged SST_N and SST_S datasets and the NAO_Gib and NAO_Azo indices confirms this finding (Fig. 2a). Both NAO indices exhibit the same reaction pattern although the relationship between SST and the NAO_Azo index is stronger than that of SST/NAO_Gib. Only within the period 1915–1929 significant NAO_Gib/SST_S correlations occur (Fig. 2a) and in general all NAO and SST datasets show a strong statistical relationship in that period. This period is nearly completely synchronous with the period of low variability (1916–1927) as described above. The finding is an initial indication that the reduced weather-type sensitivity is a result of the complex interaction and the nonstationary relationship between NAO and multidecadal variations in North Atlantic Sea Surface Temperature (SST) often referred to as Atlantic Multidecadal Variability (AMV)^50^ or the Atlantic Multidecadal Oscillation (AMO)^51^. The modes of AMO and NAO persisting over the last centuries and their footprints can be observed in local to global climate^52^,^53^,^54^ affecting even glacier mass balances^55^. The detrended SST anomaly in the North Atlantic referred to as the AMO^51^ has a nonstationary relation with the NAO^56^ and both are linked with the Atlantic Meridional Overturning Circulation (AMOC)^57^. In the negative AMO phase the NAO is strongly negatively correlated with SST. In contrast, the NAO index is weakly correlated in the positive AMO phase and negative (positive) AMO phases coincide with positive (negative) NAO phases^56^. The strongest dependencies between NAO and SST can be found in spring which is in consequence the investigated season. This NAO/AMO reaction pattern is confirmed by our findings that during the period of reduced weather-type sensitivity the NAO index is positive, while AMO is negative and the statistical relationship between both is negative (Fig. 2a up to *r* = −0.74 for 1916).

To demonstrate that SST is the trigger for the reduced weather-type sensitivity of the tree-ring width network, a mean SST time series was calculated based on the standardised SST_N and SST_S. To get a finer resolution and to narrow down the periods with SST response a 17-yr moving window is used to compute the correlation between the mean SST time series and the mean tree-ring chronology. For the complete investigation period 1891–1990 the correlation coefficient between them is *r* = 0.26 (*p* < 0.01), but in a 17-yr moving correlation window only two longer periods 1923–1932 and 1968–1975 show significant correlation coefficients (*p* <= 0.05) especially in 1925 and 1926 (*r* > 0.84, *p* < 0.0001, Fig. 2c).

The observed SST sensitivity of the tree-ring network first observed for 1923 can be seen as the reason for the already described decline in weather-type sensitivity in the beginning of the 20^th^ century because even the first correlation window 1891–1925 (1908) used for Fig. 2b has a common overlap with the correlation window used for 1923 (Fig. 2c correlation window 1915–1931). SST/tree-ring relationship increases until 1926 whereas weather-type sensitivity of the tree-ring network decreases. The observed shift between Fig. 2b,c is mainly due to the smaller correlation window used for Fig. 2c.

To confirm this finding we computed the moving correlation between tree-ring chronologies and the SST adjusted ACTI time series (ACTI adjustments only performed in period with significant tree-ring/SST relationships; see Supplementary). The results indicate stable and stronger statistical relationships even in the beginning of the time series in 1908. Therefore it can be concluded that in the beginning of the 20^th^ century the weather-type sensitivity of the tree-ring width network decreases, whereas a significant statistical relationship with SST occurs. At the end of the time series (1971) AMO is again in its negative phase but no distinct SST/NAO correlations are observable only the relationship between NAO_Azo and SST_S (Fig. 2a) slightly increases. The objective weather-type classifications, which are more sensitive to scale bias (early response than GWL C29 H/B), show a decreasing relationship for that period. In contrast, the result for GWL C29 H/B classification indicate stable statistical relationships. Additionally, again significant relationships between SST and tree-ring occur (Fig. 2c) but less pronounced as observed before. Consequently in the end of the time series presented in Fig. 2, it can be seen that weaker SST/NAO and SST/tree-ring relationships lead to small reduction in weather-type sensitivity. Only periods exhibiting distinctly negative SST/NAO correlations (e.g. NAO_Gib/SST_S correlations are only significant during the period with reduced weather-type sensitivity) can seriously affect a weather-type classification ensemble.

**Figure 3:**
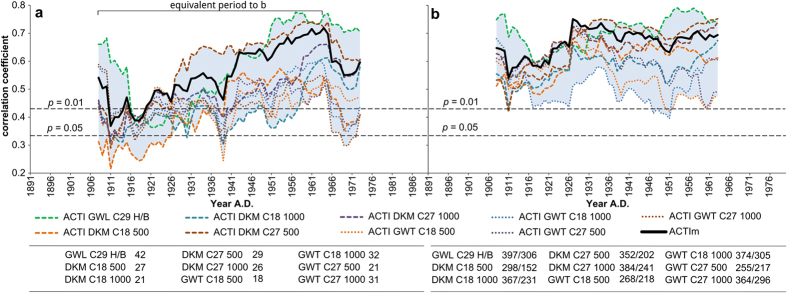
Moving correlation analysis between ACTI and tree-ring chronologies. A 35-year moving correlation window is used. For comparison purpose the curves from tree-ring dataset-1 (**a**) and dataset-2 (**b**) are shown. Common investigation period between (**a**) and (**b**) is 1891–1980. For each weather-type classification the number of chronologies with significant weather-type response and additionally for (**b**) the number of chronologies which contributed to the first principal component are depicted.

To verify the results shown in Fig. 2b we computed for all weather-type classifications based tree-ring dataset-2 ACTI time series. For each classification an unrotated principal component analysis (PCA) was used to compute the first principal component based on the ACTI time series. All ACTI time series which contribute significantly to the first principal component (*p* <= 0.05) and their associated tree-ring width chronologies were used to compute a mean curve. Hence the PCA is only applied to group ACTI and tree-ring width chronologies according to their weather-type response and to extract the common signals from the tree-ring network. For tree-ring dataset-2 649 out of 726 chronologies showed at least for one weather-type classification a significant weather-type response for spring, for dataset-1 49 out of 50 chronologies. For each weather-type classification and for both tree-ring datasets the number of chronologies with significant weather-type response for spring are shown in Fig. 3.

The moving correlation analysis (35-year window) between the ten ACTI time series (including ACTIm,) and the ten mean tree-ring chronologies dived from tree-ring dataset-2, indicates similar fluctuations in weather-type sensitivity (Fig. 3b) as already presented in Figs 2b and 3a in the beginning of the 20^th^ century. Even though a larger dataset (more chronologies, larger spatial domain) and different calibration years for the weather-type procedure were used, the curves Fig. 3 show a similar curve progression in the period with reduced weather-type sensitivity. The decline in statistical relationship especially in 1911 and 1916 are still observable as well as the reduced variability within the curves in the same period as described before (Fig. 2b). It is obvious that the relationship between ACTI time series and tree-ring width chronologies is stronger than for tree ring dataset-1 (see Fig. 3). This finding is mainly due to the larger dataset used for this analysis leading to a stabilised variance. Secondly, the spatial domain of the observed scale bias is unknown. Due to the large spatial domain of tree-ring dataset-2 (Europe, Africa 30–70 °N/10 °W-40 °E) it is extremely likely that NAO and AMO have a different impact than on tree-ring dataset-1 (Central Europe).

## Discussion

The dependencies between tree-ring chronologies and climate parameters are not always stable over time^22^,^23^ and trees growing under temperate climate conditions are often sensitive to different climate parameters^35^. Consequently for climate reconstructions often trees are used which grow under extreme environmental conditions e.g.^1^,^2^. The observed impact of the scale bias effect on the weather-type sensitivity of the tree-ring networks is more than a simple instability which is induced by changes in dependency between single climate parameters. The lower variability in the curves shown in Figs 2b and 3b and the reduced weather-type sensitivity in the beginning of the 20^th^ century are within a period with stronger NAO/SST (Fig. 2a) and tree-ring/SST (Fig. 2c) relationships. This finding is supported by the SST-adjusted ACTI time series indicating stable statistical relationships with the tree-ring chronologies (Supplementary Fig. S4). In consequence, our result indicate that phenomena in superordinate scales of the climate system can impact even the sensitivity of large proxy networks. Weather types are the leading factor for several meteorological elements^16^ and can explain the climate signal preserved in tree-ring chronologies more comprehensively^12^,^13^,^14^. In consequence, a shift, for instance from temperature sensitivity to precipitation sensitivity can not explain the observed decrease in weather-type sensitivity for the whole weather-type ensemble and in both tree-ring networks. A common trigger is needed, such as NAO and AMO to impact large proxy networks. The climate sensitivity of tree-rings can appear unstable, but in reality this is only caused by predictor sets which are incomplete and do not consider interdependencies between the predictors. Therefore as shown in Fig. 2 the influence of the different scales of the climate system on the climate signal in tree-rings is not constant over time. We have to be aware that especially reconstructions, which do not consider interdependencies between the scales of the climate system, can be seriously biased by this effect. For instance NAO sensitive tree-ring chronologies which are used for a NAO reconstruction are from a theoretical point of view, less vulnerable. The NAO sensitive tree-ring chronologies do not react directly to pressure but rather to a subset of climate variables which is driven by the state of the NAO. In consequence a change in dependency with a single climate variable does not lead inevitably to a drop in NAO sensitivity.

The detected nonstationarities between Australasian climate stations, El Niño-Southern Oscillation (ENSO) and Southern Annular Mode (SAM) demonstrate the need to quantify the effect of nonstationarities in paleoclimate reconstructions^58^. In contrast to the Australasian climate stations, our results indicate that the scale bias does not lead to a reduced statistical relationship or nonstationarities between the proxies; it can be understood as a scale dependent change in climate/growth relationships. Other factors, which may have led to the reduced weather-type sensitivity, such as simulation settings, tree-ring detrending, or an insufficient tree-ring dataset can be excluded.

In fact there is a certain within-type variation within each single weather-type^30^. Consequently we applied a weather-type ensemble, utilised a well verified tree-ring networks and used the 20^th^ Century Reanalysis dataset which is declared to be homogeneous^60^ for the North-Atlantic European region, to compute the weather-type classifications. Furthermore the results show that the statistical dependencies between ACTI and climate dataset are stable (Fig. 1e).

Our study gives evidence that more attention is needed to understand scale effects in climate reconstructions and raises the question how uncertain due to scale biases climate reconstructions are. Further research is needed to understand how processes or phenomena on different space and time scales of the climate system interact and which underlying processes or phenomena in the climate system, besides NAO and AMO, can cause changes in climate sensitivity of proxies.

Based on our results we conclude and state the following theses: Multiproxy networks are vulnerable to scale biases not only due to their tree-ring dominance, but it is also extremely likely that other climate proxies such as corals lake and sea sediments are affected as well. To reduce uncertainties in climate reconstructions the interdependencies between the processes and phenomena which act on different scales of the climate system must be considered to a greater extent. Our results confirm the need to consider ocean-atmosphere interaction^3^ and their impact on climate reconstructions more carefully. The effect of scale biases on the climate sensitivity of the proxies is discontinuous but periodically (here NAO/AMO interrelation which follows the AMO period of ~60 to 80-years^52^) and can therefore impact the high (as shown here) to low-frequency spectrum in the proxy records. The found spectral biases^25^ in climate reconstructions which lead to on overestimation of low-frequency signals may be caused by scale biases. The reason for spectral biases^25^ is not completely understood and we could not prove this relationship, but the scale bias effect can cause similar biases as reported. Based on our results the impact of phenomena on superordinate scales of the climate system e.g. NAO, AMO, PDO, or El Niño must be taken into account more thoroughly.

Examining scale bias from an inverse perspective leads to the conclusion that it hinders the assessment of the influence of past climate conditions on climate proxies. As a result, the estimation of the influence of scale bias within future climate scenarios is even more complicated as it relies on assumptions that are derived from physical observations. To improve the understanding of interdependencies and feedbacks in the climate/earth system the role of scale effects must be re-assessed.

## Additional Information

**How to cite this article**: Schultz, J. A. *et al.* Sensitivity of proxies on non-linear interactions in the climate system. *Sci. Rep.* **5**, 18560; doi: 10.1038/srep18560 (2015).

## Supplementary Material

Supplementary Information

Supplementary Dataset S1

Supplementary Dataset S2

Supplementary Dataset S3
