# Supplementary material for: Sensitivity of proxies on non-linear interactions in the climate system
Source: Sci Rep. 2015 Dec 21;5:18560. doi: 10.1038/srep18560 (PMC4685260; doi:10.1038/srep18560)
Supplement: Supplementary Information [file srep18560-s1.pdf]

Supplementary Information

Sensitivity of proxies on non-linear interactions in the climate system

Authors: Johannes A. Schultz,[1]* Christoph Beck,[2] Gunter Menz,[1] Burkhard Neuwirth,[3] Christian A. Ohlwein,[4] Andreas Phillipp[2]

Affiliations:

[1]Department of Geography, University of Bonn, 53115 Bonn, Germany.

[2]Department of Geography, University of Augsburg, 86159 Augsburg, Germany.

[3]DeLaWi Dendro Lab Windeck, 51570 Windeck, Germany.

[4] Hans-Ertel-Centre for Weather Research, Meteorological Institute, University of Bonn, 53121 Bonn, Germany

*Correspondence to: schultz@giub.uni-bonn.de.

S1 Tree-ring data

Tree-ring dataset-1 consist out of 21 beech and 29 oak chronologies which were taken from an already published tree-ring width network [1, 2]. The detrended site chronologies (see Methods) can be found in the dataset S1 Chronos. The values of the statistical parameters, expressed population signal (EPS) [3], which indicates how well the site chronology estimates a theoretically infinite population and interseries correlation (Rbar) [4] – averaged correlation between tree-ring series – are shown with additional meta information in Table S1. As indicated in Table S1 all chronologies cover the complete investigation period 1891-1990.

Tree ring dataset-2 is extensively described and the datasets are available in the Supplementary of Babst 2013 et al. [5] We only used chronologies which cover the period 1881-1980. In consequence only 726 chronologies were used. In dataset S2 Inventory all used chronologies are listed. 42 chronologies are common to both datasets.

S2 General remarks concerning ACTI time series (tree-ring dataset-1)

In total nine different weather-type classifications were applied. In consequence ten ACTI time series including the ACTIm, which was derived by averaging the nine ACTI time series, were computed.

To investigate common signals between the ten ACTI time series and gridded pressure data we performed a spatial correlation analysis (Fig. S1). In addition the pairwise correlations was calculated, for the mean tree-ring width chronologies computed for each weather-type classification and for the ACTI time series (Fig. S3). Pairwise correlation coefficients indicate no large differences among the tree-ring chronologies (Fig. S3a). In contrast, the correlation coefficients computed between the ACTI time series indicate a weak but still significant statistical relationship (Fig. S3b).

It can therefore be concluded that the differences observed among the curves graphed in Fig. 2b are mostly due to variance among the ACTI time series. Despite these differences, spatial correlation analyses of the 10 ACTI time series and gridded pressure data show a distinct common pattern (Fig. S1). A negative correlation with pressure in the North Sea and Baltic Sea regions is present for all ACTI time series. Low pressure in those areas is directly affecting the climate conditions at the relevant tree-ring sites. Statistical relationships observed outside of the study area, such as for the Greenland/North America region reflects the common large-scale interrelation within the west wind drift.

Considering the entire 1891-1990 time period it is clear that the statistical relationship between the tree-ring chronologies and ACTI time series shows a large variability among and within the curves (Fig. 2b). These results indicate that the weather-type classifications which were implemented here are not equally capable to investigate climate/growth relationships. The reduced variability between the curves and the decline in weather-type sensitivity (Fig. 2b) is an indication for a common trigger in the beginning of the 20th century. This finding is also supported by the results presented in Fig. S4 which displays the moving correlation between the SST-adjusted ACTI time series and the tree-ring width chronologies. To calculate ACTI-adjusted we used the relationships between NAO and SST (Fig. 2a), and the relationships between SST and tree-ring chronologies (Fig. 2c) which allow due to the smaller correlation window a finer resolution, to narrow down the period with reduced weather-type sensitivity to 1917-1934. This period (1917-1934) is synchronous with the correlation windows used for 1925 and 1926 (Fig. 2c), which showed the strongest statistical dependencies between SST and the mean tree-ring chronology.

For the period 1917-1934 the mean standardised SST time series (Fig. 2 caption) is added to the standardised ACTI time series. Consequently all ACTI time series received the same SST correction and are modified only in the period 1917-1934. We observed that this empirical adjustment of the 1917-1934 years eliminates the decline in weather-type sensitivity. This is indicated for ACTIm by the adjusted curve (plotted as the blue double-line) shown in Supplementary Fig. S4a. The impact of the SST adjustment on all ACTI time series is shown in Supplementary Fig. S4b.

Adding an SST influence to the entire ACTIm time series (1891-1990) leads to a reduced statistical relationship expressed by a correlation coefficient of $r = 0.47$ vs. $r = 0.58$ (1891-1990). In this context,

comparing the results from the moving correlation analyses between tree-ring chronologies and original ACTI and modified ACTI time series (Supplementary Fig. S4a), two aspects can be concluded:

1. The ACTIm and ACTIm-adjusted curves shown in Supplementary Fig. S4a are quite similar in shape. The adjusted curve is apparently simply shifted to a higher level of statistical relationship.

2. Adding an SST influence to all ACTI time series for the 1917-1934 period produces the same positive effect (Fig. S4b).

Table S1. Inventory of the tree-ring width network (dataset-1). FASY = *Fagus sylvatica* (common beech), QUSP = *Quercus sp*., (oak), QUPE = *Quercus petraea* (sessile oak), QURO = *Quercus robur* (pedunculate oak)

ID	Lat/Long	Species	Elev.	Rbar	EPS	Period	ID	Lat/Long	Species	Elev.	Rbar	EPS	Period
1	49.525/10.522	QUSP	415	0.62	0.97	1785-1990	26	50.680/7.041	QURO	165	0.57	0.95	1848-2006
2	48.265/12.957	FASY	465	0.39	0.93	1800-1995	27	50.609/6.457	FASY	530	0.50	0.94	1834-2004
3	50.201/9.563	FASY	465	0.41	0.94	1860-1995	28	50.606/6.490	FASY	470	0.63	0.96	1863-2004
4	50.201/9.563	QUPE	465	0.59	0.97	1857-1995	29	50.681/6.279	QUPE	460	0.54	0.93	1810-2004
5	50.199/9.560	FASY	470	0.49	0.95	1864-1995	30	50.624/6.399	QUPE	400	0.50	0.93	1813-2004
6	50.199/9.560	QUSP	470	0.50	0.95	1864-1995	31	50.571/6.361	FASY	490	0.57	0.94	1820-2004
7	51.169/8.962	FASY	340	0.55	0.90	1798-2005	32	50.571/6.360	QUPE	500	0.49	0.92	1847-2004
8	51.170/8.967	FASY	310	0.51	0.94	1837-2005	33	50.572/6.361	FASY	480	0.56	0.94	1828-2004
9	51.171/8.968	QUPE	290	0.45	0.93	1839-2005	34	50.729/8.119	FASY	500	0.53	0.94	1848-2005
10	51.167/8.958	FASY	420	0.66	0.97	1798-2005	35	50.727/8.118	QUPE	450	0.50	0.90	1750-2005
11	51.156/9.084	QUPE	350	0.57	0.93	1844-2005	36	50.866/8.225	FASY	440	0.53	0.94	1856-2005
12	51.157/9.084	QUPE	365	0.60	0.90	1863-2005	37	50.866/8.227	QUPE	440	0.65	0.96	1857-2005
13	51.156/9.077	QUPE	390	0.59	0.93	1851-2005	38	50.927/6.419	QURO	105	0.57	0.95	1835-2005
14	49.616/7.922	QUPE	500	0.60	0.93	1840-2005	39	50.917/6.424	QURO	103	0.49	0.91	1766-2005
15	50.044/7.071	QUPE	270	0.63	0.94	1831-2005	40	50.792/6.844	FASY	155	0.52	0.91	1871-2005
16	50.297/7.007	QUPE	480	0.53	0.91	1832-2005	41	50.792/6.844	QURO	155	0.60	0.94	1862-2005
17	50.297/7.007	FASY	480	0.47	0.90	1839-2005	42	51.230/7.111	QUPE	260	0.61	0.94	1863-2005
18	49.880/7.583	QUPE	500	0.50	0.93	1812-2005	43	51.674/6.370	FASY	50	0.40	0.91	1859-2008
19	49.864/7.235	QUPE	520	0.57	0.94	1661-2005	44	51.674/6.364	FASY	60	0.54	0.93	1841-2008
20	49.304/7.747	QUPE	400	0.56	0.93	1708-2005	45	51.102/8.022	FASY	455	0.54	0.94	1864-2005
21	49.262/7.814	QUPE	475	0.55	0.94	1717-2005	46	51.447/8.130	FASY	360	0.42	0.92	1805-2006
22	49.055/7.643	QUPE	390	0.59	0.93	1713-2005	47	50.399/10.593	FASY	460	0.61	0.94	1855-1994
23	50.670/7.247	FASY	340	0.55	0.94	1863-2004	48	50.401/10.594	FASY	520	0.44	0.90	1848-1994
24	50.671/7.047	QURO	185	0.54	0.92	1832-2005	49	50.399/10.596	QURO	460	0.53	0.94	1804-1994
25	50.670/7.047	QURO	185	0.54	0.93	1850-2005	50	50.399/10.593	QURO	420	0.67	0.96	1799-1994

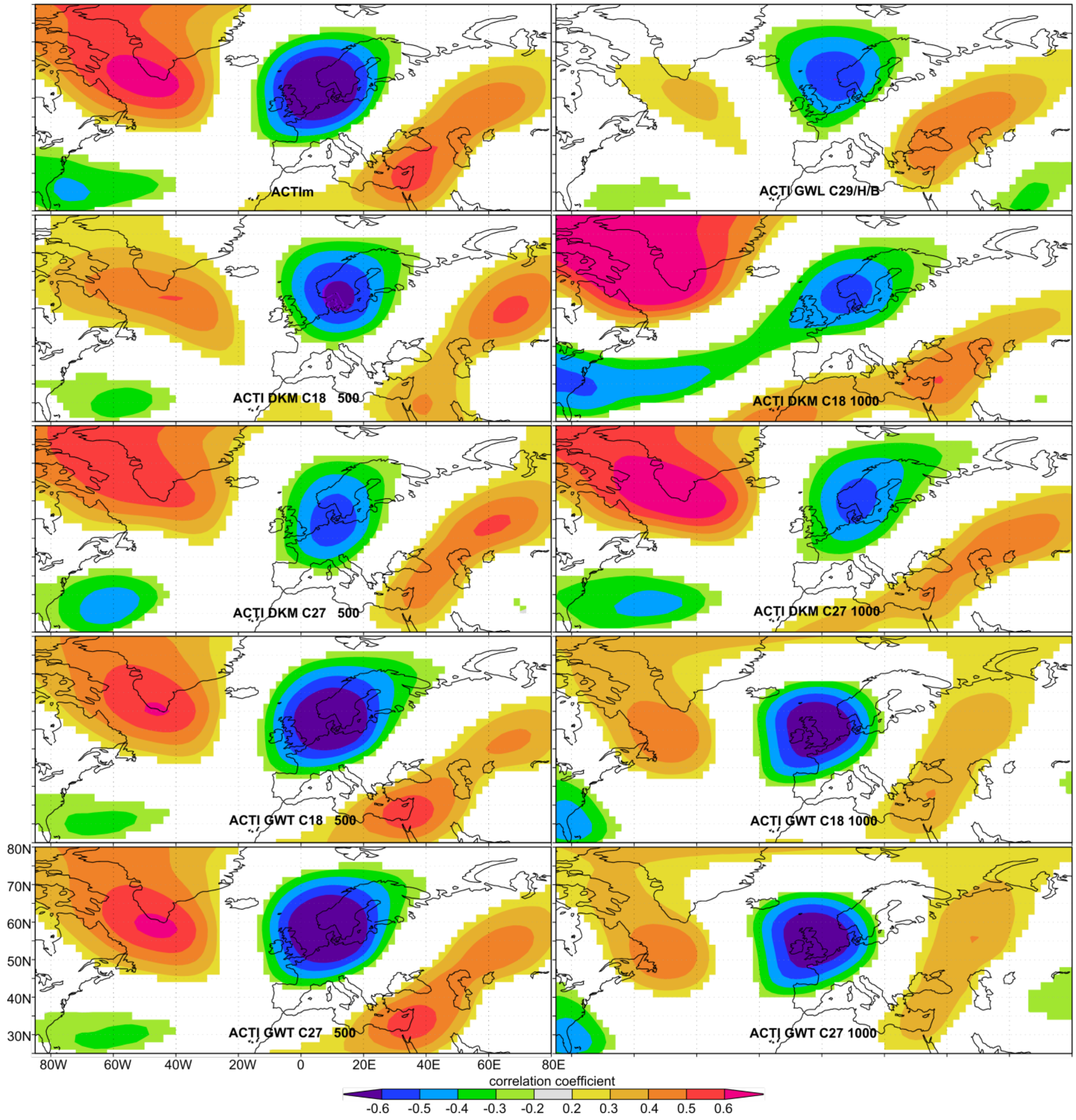

Figure S1: Spatial correlation between ACTI time series and 20th Century reanalysis pressure data [6] (500 hPa) (Pearsons correlation coefficient) computed with the Climate Explorer [7] web application a service of the Royal Netherlands Meteorological Institute and arranged with Inkscape. Coloured areas reflect significance (*p* <= 0.05). All datasets were high pass filtered (year-on-year differences) and all calculations are based on the period 1891-1990.

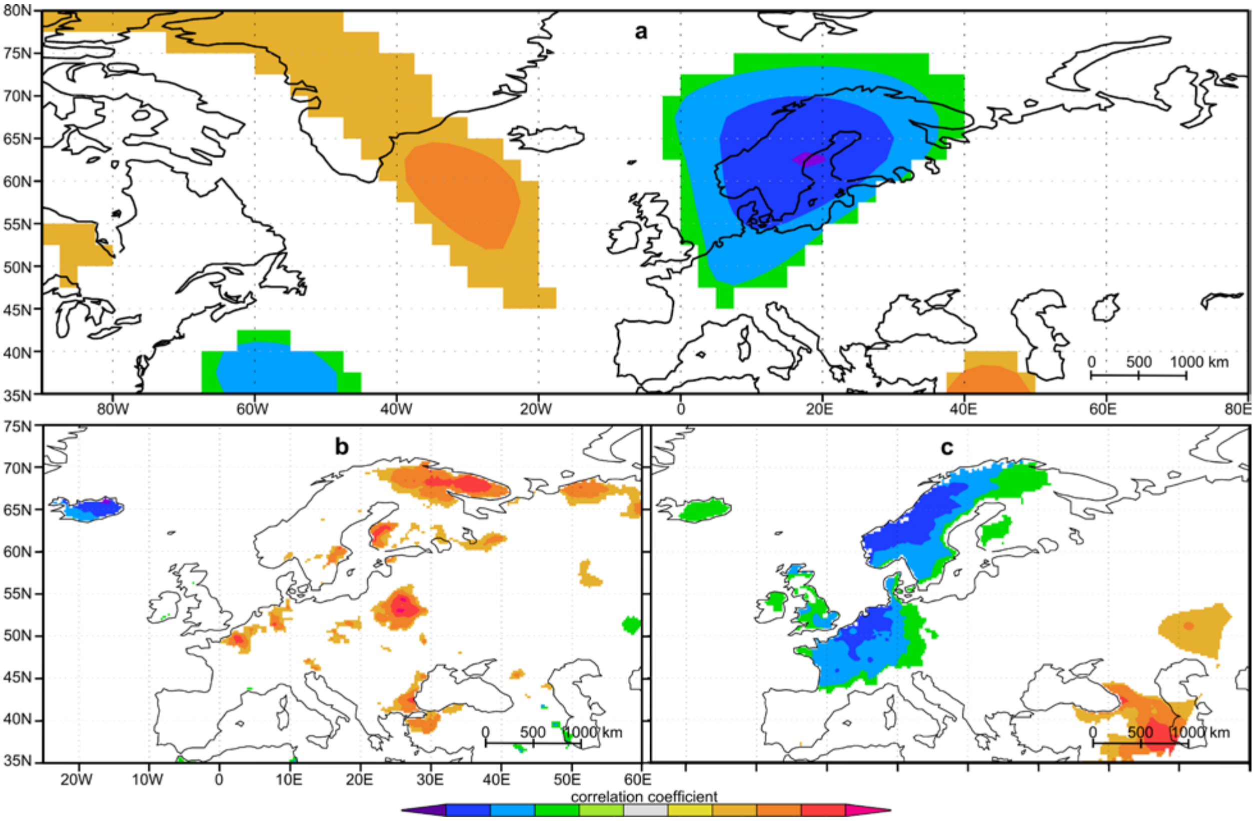

Figure S2: Spatial correlation between tree-ring chronologies and gridded climate datasets (a-c), (a) NCEP/NCAR Reanalysis 1, pressure [8] (500 hPa), (b) E-OBS 10, precipitation [9] (c) E-OBS 10, maximum temperature [9]. The maps were generated with the Climate Explorer [7] web application a service of the Royal Netherlands Meteorological Institute and arranged with Inkscape. Coloured areas reflect significance (p <= 0.05). Pearson correlation coefficients were computed based on the 1951-1990 period common among the datasets (a-c). All calculations are based on high pass filter time series (year-on-year differences).

	TRW / ACTI GWL C29 H/B	TRW / ACTI DKM C18 500	TRW / ACTI DKM C18 1000	TRW / ACTI DKM C27 500	TRW / ACTI DKM C27 1000	TRW / ACTI GWT C18 500	TRW / ACTI GWT C18 1000	TRW / ACTI GWT C27 500	TRW / ACTI GWT C27 1000	TRW / ACTIm
TRW / ACTI GWL C29 H/B		0.45	0.34	0.54	0.51	0.48	0.48	0.51	0.46	0.64
TRW / ACTI DKM C18 500	0.94		0.33	0.60	0.47	0.57	0.45	0.62	0.43	0.67
TRW / ACTI DKM C18 1000	0.84	0.79		0.61	0.80	0.65	0.69	0.67	0.64	0.80
TRW / ACTI DKM C27 500	0.97	0.91	0.92		0.68	0.67	0.57	0.69	0.52	0.80
TRW / ACTI DKM C27 1000	0.93	0.90	0.95	0.96		0.68	0.63	0.71	0.58	0.83
TRW / ACTI GWT C18 500	0.92	0.86	0.94	0.96	0.97		0.63	0.96	0.59	0.87
TRW / ACTI GWT C18 1000	0.96	0.89	0.93	0.98	0.96	0.98		0.70	0.99	0.86
TRW / ACTI GWT C27 500	0.96	0.89	0.89	0.97	0.96	0.97	0.96		0.66	0.91
TRW / ACTI GWT C27 1000	0.95	0.87	0.92	0.98	0.95	0.97	1.00	0.95		0.83
TRW / ACTIm	0.97	0.92	0.94	0.99	0.98	0.98	0.99	0.98	0.99	

(a: lower triangle; b: upper triangle)

Figure S3: Correlation matrix for ACTI and the tree-ring chronologies. Pearson's correlation coefficients computed between (**a**) the mean tree-ring chronologies and (**b**) between the ACTI time series. All calculations are based on the period 1891-1990. TRW and ACTI time series can be found in dataset S3 TRW_ACTI.

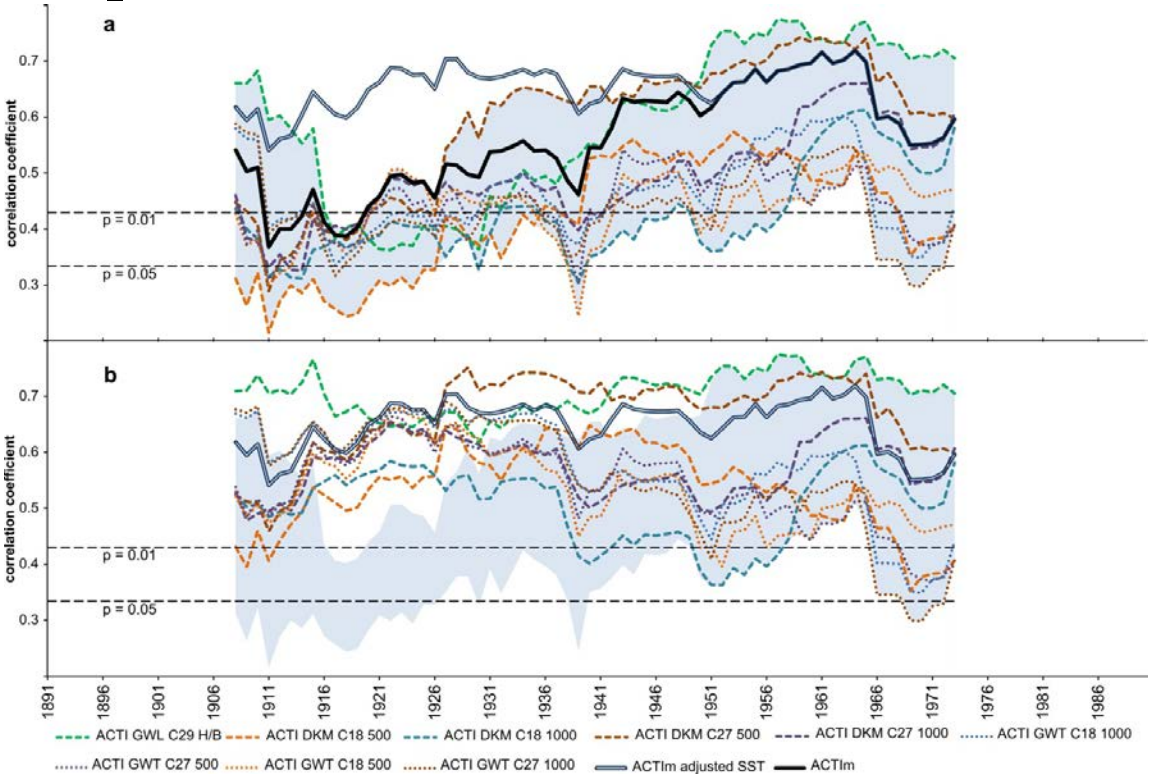

Figure S4: Moving correlation in a 35-yr window between ACTI and tree-ring chronologies. (**a**) original ACTI time series including ACTIm-SST adjusted (plotted as the blue double-line) are shown and for (**b**) SST adjustment is applied to all ACTI time series. For (**a**) and (**b**) Pearson correlation coefficient is used.

References

[1] Schultz, J. A., Neuwirth, B. A new atmospheric circulation tree-ring index (ACTI) derived from climate proxies: Procedure, results and applications. *Agric. For. Meteorol.* **164,** 149-160 (2012).

[2] Neuwirth, B. Schweingruber, F. H., Winiger, M. Spatial patterns of central European pointer years from 1901 to 1971. *Dendrochronologia* **24,** 79-89 (2007).

[3] Wigley, T. M. L., Briffa, K.R., Jones, P.D. On the average value of correlated time series, with applications in dendroclimatology and hydrometeorology. *J. Climate* **23,** 201-213(1984).

[4] Cook. E. R., Kairiukstis, L.A. Methods of Dendrochronology: *Applications in the Environmental Sciences*, (Kluwer, Dordrecht NL) pp. 394 (1990).

[5] Babst, F. *et al*. Site- and species-specific responses of forest growth to climate across the European continent, Glob. Ecol. Biogeogr., 22, 706–717 (2013).

[6] Compo, G. P. *et al*. The Twentieth Century Reanalysis Project, Q. J. R. Meteorol. Soc. 137, 1–28 (2011).

[7] van Oldenborgh, G. J. *et al*. Western Europe is warming much faster than expected, Clim. Past. 5, 1–12 (2009).

[8] Kalnay, E. *et al*. The NCEP/NCAR 40-year reanalysis project, Bull. Am. Meteorol. Soc.77, 437–471 (1996).

[9] Haylock, M. R. *et al*. A European daily high-resolution gridded data set of surface temperature and precipitation for 1950-2006, J. Geophys. Res.-Atmos. 113 D20119 (2008).
